# Supplementary material for: Apoptotic bodies in phytoplankton suggest evolutionary conservation of cell death mechanisms
Source: Nat Commun. 2025 Sep 25;16:8427. doi: 10.1038/s41467-025-63956-4 (PMC12462507; doi:10.1038/s41467-025-63956-4)
Supplement: Supplementary file 1 — Supplementary Information [file 41467_2025_63956_MOESM1_ESM.pdf]

Supplementary Information

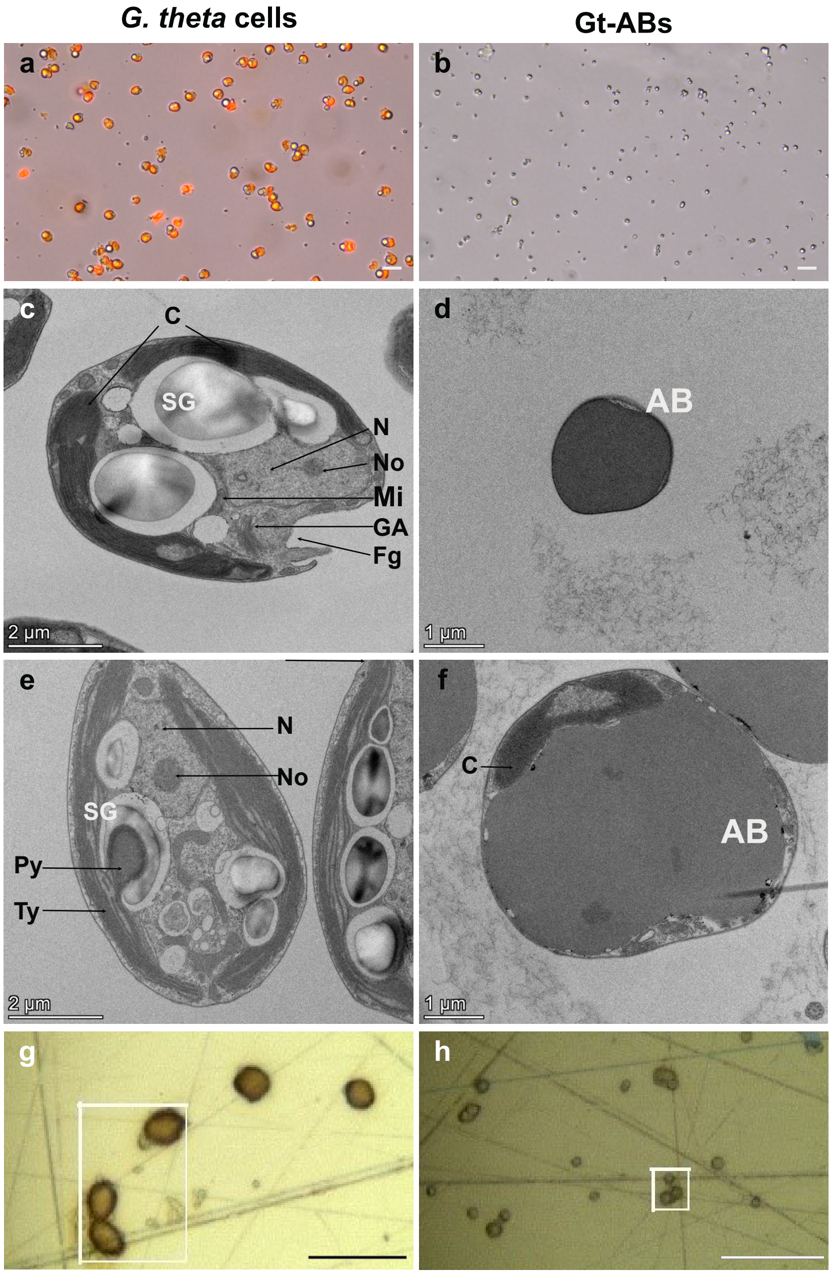

Supplementary Figure 1. Comparison of size and complexity, *G. theta* cells versus Gt-ABs. Fluorescence microscopy overlay images of *G. theta* cells displaying chlorophyll fluorescence in red **(a)** and Gt-ABs, which lack chlorophyll **(b).** Scale bars correspond to 10 µm. TEM images of healthy *G. theta* cells growing in the exponential phase showing typical vegetative cell morphology **(c, e)** including N—nucleus, No—nucleolus, C—chloroplast, GA—Golgi apparatus, Mi—mitochondrion, Fg—flagella and H shaped chloroplast with Py—starch-cap encased pyrenoid, Ty—thylakoid membranes and SG—starch granule In comparison small Gt-ABs (2 µm) **(d)** and large Gt-ABs (3-5 µm) **(f)**. Confocal RAMAN spectroscopy images (scale bars correspond to 20 µm) of healthy *G. theta* cells growing in the exponential phase with regular pigmentation **(g)** in contrast to Gt-ABs **(h)** of the death phase lacking pigments. Gt- ABs images (right column, panels B, D, F, H) correspond to purified and sorted populations of Gt-ABs. The images are representative of 2 independent experiments, for fluorescence microscopy and RAMAN spectroscopy, and 6 independent experiments, for TEM imaging, with consistent morphology observed.

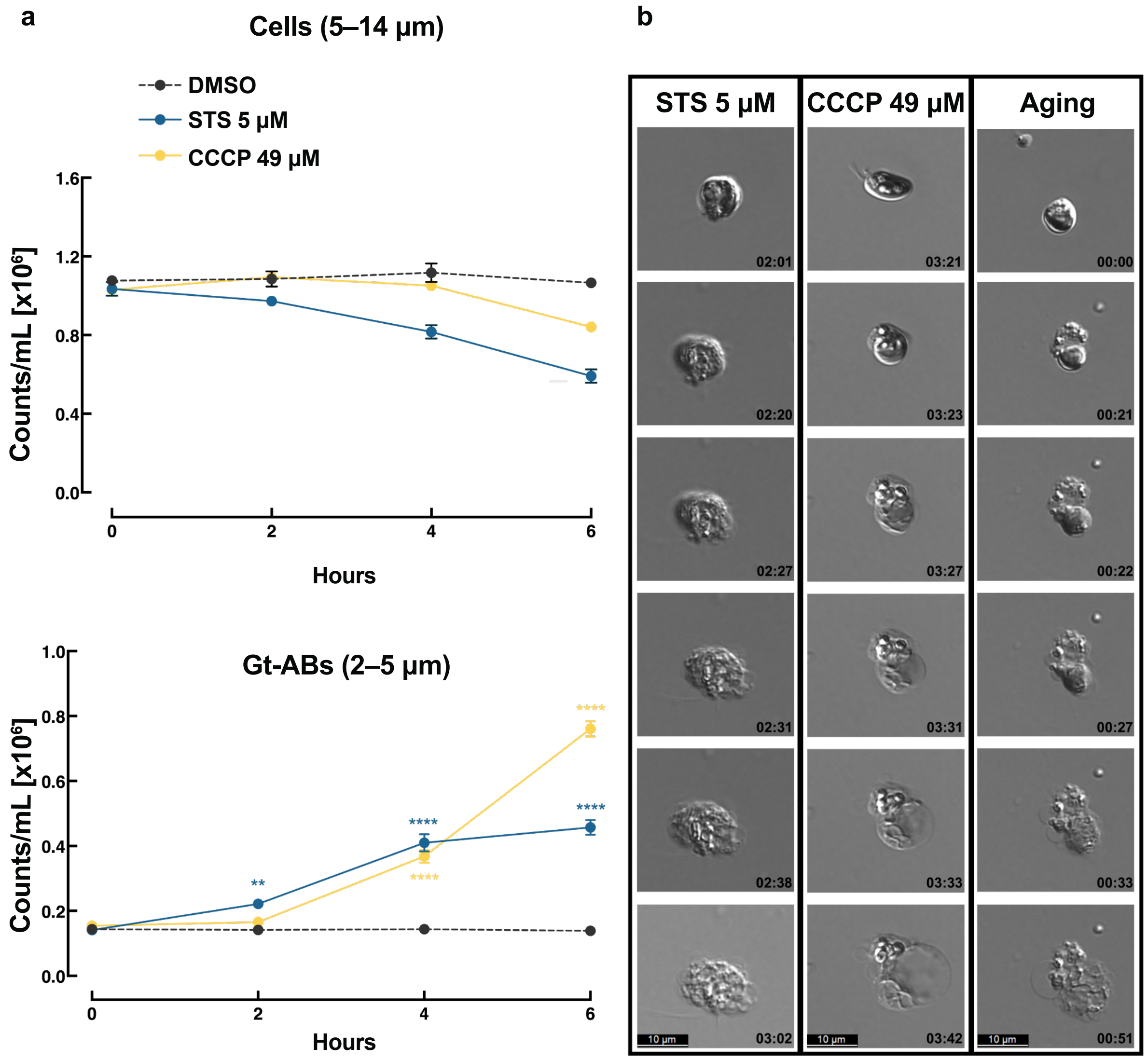

Supplementary Figure 2. Pharmacological induction of apoptosis in *G. theta* cultures. a. Number of cells and Gt-ABs upon apoptosis induction in the cell population using staurosporine (STS, 5 µM) or carbonyl cyanide m-chlorophenyl hydrazone (CCCP, 49 µM). Dimethyl sulfoxide (DMSO (vehicle), 0.115%) was used as a negative control. **b.** Timelapse-DIC images of *G. theta* cells growing in the exponential culture phase exposed to 5 µM STS or 49 µM CCCP; the time stamp on the images (hh:mm) indicates the time from the initial addition of the apoptosis inducers. In comparison natural aging *G. theta* cells (grown for 58 days) are shown without addition of pharmacological treatment. The time stamp for the untreated ageing cells indicates the time from the starting point of the apoptotic process. Scale bar corresponds to 10 µm. Representative micrographs from 3 independent experiments are shown. For a. data are presented as mean values +/- SEM of biologically independent samples (n=3). The number of Gt-ABs produced after pharmacological treatment were compared to negative control at each time point using two-way ANOVA with Dunnett's multiple comparison test (***P* = 0.0028, *****P* <0.0001). Non-significant comparisons with *P* >0.05 are not shown. Source data are provided as a Source data file.

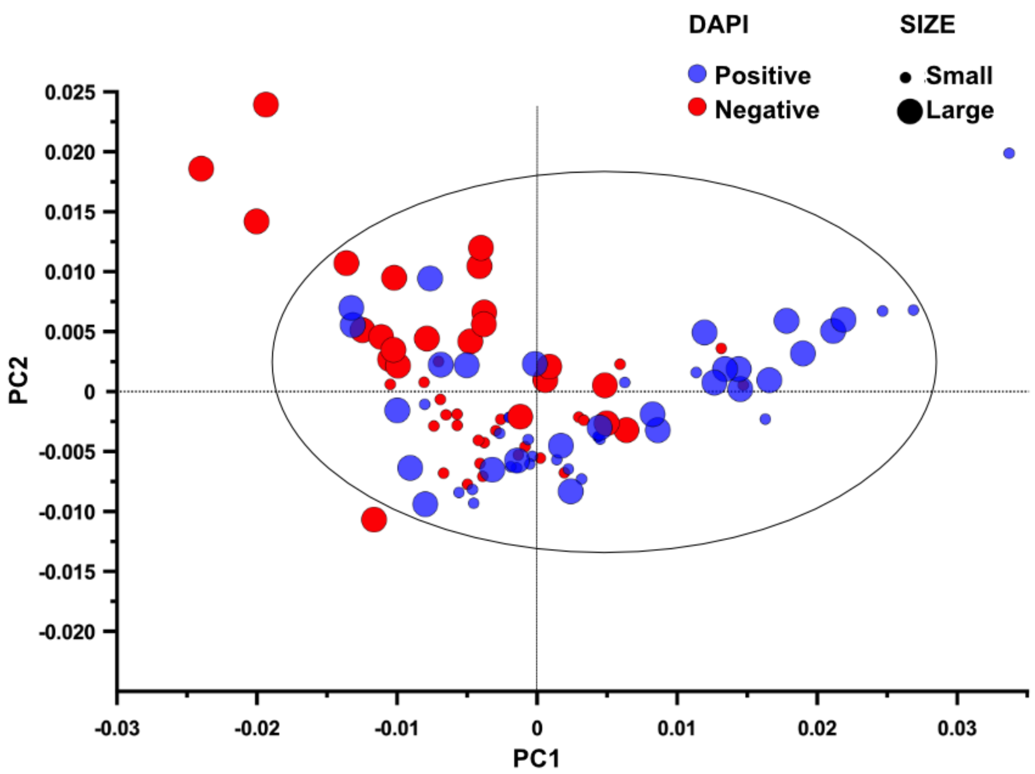

Supplementary Figure 3. Multivariate analysis of all RAMAN spectra of Gt-ABs. Scatter plot of the Principal Components Analysis (PCA) scores of the total number of spectra (N=99). Each circle represents one spectrum, and the ellipsoid represents the 95% confidence interval. Principal component 1 (PC1) accounted for 37.5% of the total variance and PC2 for 14.6%. Large (3–5 μm) and small (2 μm) Gt-ABs subpopulations cluster in different groups according to their sizes. Variation within the spectra of large Gt-ABs was based on their DAPI-positive or negative fluorescence. Most spectra of small Gt-ABs clustered together indicating no difference in the composition of DAPI-negative and positive small Gt-AB subpopulations. Source data are provided as a Source data file.

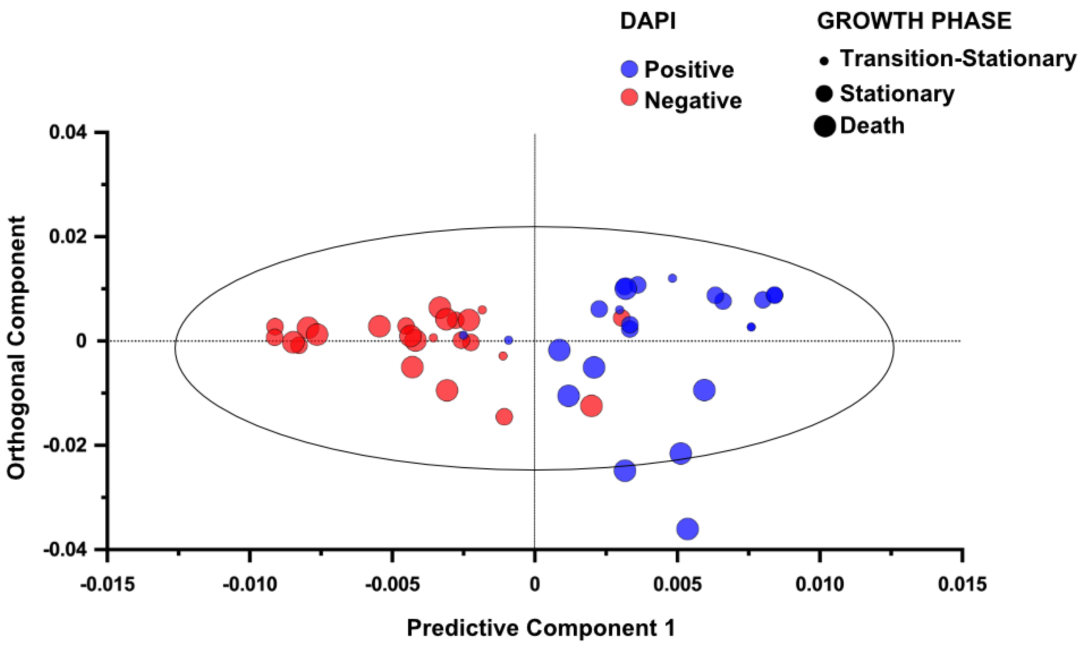

Supplementary Figure 4. Orthogonal projections to latent structures - discriminant analysis (OPLS-DA) of Raman spectra on small Gt-AB subpopulations. Scatter plot of the OPLS–DA model scores on spectra of Gt-AB subpopulations with a size of 2 µm (N=49, Q2=0.329). Each circle represents one spectrum and the ellipsoid represents the 95% confidence interval. DAPI-positive (blue) subpopulations separated from DAPI-negative ones (red); within the spectra of DAPI-positive subpopulations, two groups are observed corresponding to Gt-ABs of the stationary and the death phase, respectively. Spectra of Gt-ABs sampled on day 11 (transition to stationary phase) and days 17 and 24 (stationary phase) clustered together, indicating similar composition of these Gt-ABs. Spectra of Gt-ABs of the death phase (days 28 and 42) are well separated, indicating different composition to Gt-ABs of the stationary phase. Source data are provided as a Source data file.

Supplementary Table 1. Number of recorded events of Gt-AB subpopulations. Four subpopulations of Gt-ABs were identified and sorted: size 2 µm, DAPI-positive; size 2 µm, DAPI-negative; size 3-5 µm, DAPI-positive and size 3-5 µm, DAPI negative. The number of Gt-ABs was recorded as the number of events during FACS analysis. A higher amount of DAPI-negative Gt-ABs was observed in both stationary and death phases. Statistically significant differences were found for the DAPI signal (+/-) in the Gt-ABs, the growth phases (stationary and death) and the interaction of both factors. The analysis was performed using two way ANOVA for the large ABs (3-5 µm) (DAPI ****P <0.0001, growth phase ****P <0.0001, interaction ****P <0.0001) and the small ABs (2 µm) (DAPI ****P <0.0001, growth phase **P <0.0069, interaction **P <0.0090). Data are presented as mean values of biologically independent samples (n=4).

		**Day 17**		**Day 24**		**Day 28**		**Day 42**	
	**DAPI**	Negative	Positive	Negative	Positive	Negative	Positive	Negative	Positive
**Size**	**3-5 um**	28749	307	20513	622	8703	423	9303	369
	**2 um**	10490	260	17380	940	19385	626	21822	804

Supplementary Table 2. List of oligonucleotide RT-qPCR primers used in this study for the expression analysis of *G. theta* metacaspase genes.

**Type of gene**	**Gene name**	**Name of the Primer**	**Sequence (5' —3')**	**Size (bp)**	**Reference**
**Housekeeping (reference genes)**	Tubulin beta	tubb_F	CATGGTGGGATTTGCTCCCT	20	49
		tubb_R	GCATCAAACATCTGCTGCGT		
	Dynein	Dyn_F	CTGTGCAGTACGCGAGTTTG		
		Dyn_R	TCGATCGCACTCGGAGAAAC		
**Metacaspase genes**	*G. theta* metacaspase I	GtMC1_F	ATCCGTGCTTACAATGCCCA		
		GtMC1_R	GCTGAGGAACCTGACCGAAA		
	G. theta metacaspase III	GtMC2_F	TTCATGCATTACTCGGGCCA		
		GtMC2_R	CTGGTGTAGTCCACAGGCAC		
