# Supplementary material for: Apoptotic bodies in phytoplankton suggest evolutionary conservation of cell death mechanisms
Source: Nat Commun. 2025 Sep 25;16:8427. doi: 10.1038/s41467-025-63956-4 (PMC12462507; doi:10.1038/s41467-025-63956-4)
Supplement: Supplementary file 2 — Description of Additional Supplementary Information [file 41467_2025_63956_MOESM2_ESM.pdf]

Description of Additional Supplementary Files

File Name: Supplementary Data 1
Description: Peak assignments for bands with over 50% correlations in significant OPLS-DA models of Gt-ABs subpopulations. Detailed Raman features, statistical correlations and corresponding peaks from the literature are included.

File Name: Supplementary Data 2
Description: Comparison between Apoptotic Bodies (ABs) and other types of extra- or intracellular vesicles from eukaryotic and prokaryotic origin.
