# Supplementary material for: Apoptotic bodies in phytoplankton suggest evolutionary conservation of cell death mechanisms
Source: Nat Commun. 2025 Sep 25;16:8427. doi: 10.1038/s41467-025-63956-4 (PMC12462507; doi:10.1038/s41467-025-63956-4)
Supplement: Supplementary file 5 — Reporting Summary [file 41467_2025_63956_MOESM5_ESM.pdf]

nature portfolio

Corresponding author(s): Christiane Funk, Luisa Corredor

Last updated by author(s): Aug 19, 2025

Reporting Summary

Nature Portfolio wishes to improve the reproducibility of the work that we publish. This form provides structure for consistency and transparency in reporting. For further information on Nature Portfolio policies, see our Editorial Policies and the Editorial Policy Checklist.

Statistics

For all statistical analyses, confirm that the following items are present in the figure legend, table legend, main text, or Methods section.

n/a	Confirmed	
☐	☒	The exact sample size (n) for each experimental group/condition, given as a discrete number and unit of measurement
☐	☒	A statement on whether measurements were taken from distinct samples or whether the same sample was measured repeatedly
☒	☐	The statistical test(s) used AND whether they are one- or two-sided<br>*Only common tests should be described solely by name; describe more complex techniques in the Methods section.*
☒	☐	A description of all covariates tested
☒	☐	A description of any assumptions or corrections, such as tests of normality and adjustment for multiple comparisons
☐	☒	A full description of the statistical parameters including central tendency (e.g. means) or other basic estimates (e.g. regression coefficient) AND variation (e.g. standard deviation) or associated estimates of uncertainty (e.g. confidence intervals)
☐	☒	For null hypothesis testing, the test statistic (e.g. F, t, r) with confidence intervals, effect sizes, degrees of freedom and P value noted<br>*Give P values as exact values whenever suitable.*
☒	☐	For Bayesian analysis, information on the choice of priors and Markov chain Monte Carlo settings
☒	☐	For hierarchical and complex designs, identification of the appropriate level for tests and full reporting of outcomes
☒	☐	Estimates of effect sizes (e.g. Cohen's d, Pearson's r), indicating how they were calculated

Our web collection on statistics for biologists contains articles on many of the points above.

Software and code

Policy information about availability of computer code

Data collection	Renishaw's WiRE software (version 5.6), BD FACSDiva software version 8.0.3, Multisizer 586 software (Version 3.53), Velox software v2.14.2.40
Data analysis	MCR-ALS v6c Application, using MATLAB R2023a Update 5, GraphPad 819 Prism 10.1.2 (GraphPad Software Inc.)

For manuscripts utilizing custom algorithms or software that are central to the research but not yet described in published literature, software must be made available to editors and reviewers. We strongly encourage code deposition in a community repository (e.g. GitHub). See the Nature Portfolio guidelines for submitting code & software for further information.

Data

Policy information about availability of data

All manuscripts must include a data availability statement. This statement should provide the following information, where applicable:

- Accession codes, unique identifiers, or web links for publicly available datasets
- A description of any restrictions on data availability
- For clinical datasets or third party data, please ensure that the statement adheres to our policy

All data supporting the findings of this study are available within the paper and its Supplementary Information.

Research involving human participants, their data, or biological material

Policy information about studies with human participants or human data. See also policy information about sex, gender (identity/presentation), and sexual orientation and race, ethnicity and racism.

Reporting on sex and gender	*Use the terms sex (biological attribute) and gender (shaped by social and cultural circumstances) carefully in order to avoid confusing both terms. Indicate if findings apply to only one sex or gender; describe whether sex and gender were considered in study design; whether sex and/or gender was determined based on self-reporting or assigned and methods used. Provide in the source data disaggregated sex and gender data, where this information has been collected, and if consent has been obtained for sharing of individual-level data; provide overall numbers in this Reporting Summary. Please state if this information has not been collected. Report sex- and gender-based analyses where performed, justify reasons for lack of sex- and gender-based analysis.*
Reporting on race, ethnicity, or other socially relevant groupings	*Please specify the socially constructed or socially relevant categorization variable(s) used in your manuscript and explain why they were used. Please note that such variables should not be used as proxies for other socially constructed/relevant variables (for example, race or ethnicity should not be used as a proxy for socioeconomic status). Provide clear definitions of the relevant terms used, how they were provided (by the participants/respondents, the researchers, or third parties), and the method(s) used to classify people into the different categories (e.g. self-report, census or administrative data, social media data, etc.) Please provide details about how you controlled for confounding variables in your analyses.*
Population characteristics	*Describe the covariate-relevant population characteristics of the human research participants (e.g. age, genotypic information, past and current diagnosis and treatment categories). If you filled out the behavioural & social sciences study design questions and have nothing to add here, write "See above."*
Recruitment	*Describe how participants were recruited. Outline any potential self-selection bias or other biases that may be present and how these are likely to impact results.*
Ethics oversight	*Identify the organization(s) that approved the study protocol.*

Note that full information on the approval of the study protocol must also be provided in the manuscript.

Field-specific reporting

Please select the one below that is the best fit for your research. If you are not sure, read the appropriate sections before making your selection.

- [x] Life sciences
- [ ] Behavioural & social sciences
- [ ] Ecological, evolutionary & environmental sciences

For a reference copy of the document with all sections, see nature.com/documents/nr-reporting-summary-flat.pdf

Life sciences study design

All studies must disclose on these points even when the disclosure is negative.

Sample size	No statistical methods were used to determine the sample size. The sample size (n) used for each method is stated in the manuscript.
Data exclusions	The identified population of G. theta cells with relative size larger than 5 µm and chlorophyll autofluorescence emission was excluded from sorting and was not included in the analysis, since it was out of the scope of this study.
Replication	The data originate from at least three biological replicates and two technical replicates. The experiments were reproducible. The specific replicate number for each experiment is indicated in the manuscript.
Randomization	No randomization was applied.
Blinding	No blinding was used in the present work.

Reporting for specific materials, systems and methods

We require information from authors about some types of materials, experimental systems and methods used in many studies. Here, indicate whether each material, system or method listed is relevant to your study. If you are not sure if a list item applies to your research, read the appropriate section before selecting a response.

Materials & experimental systems

n/a	Involved in the study
[x]	[ ] Antibodies
[x]	[ ] Eukaryotic cell lines
[x]	[ ] Palaeontology and archaeology
[x]	[ ] Animals and other organisms
[x]	[ ] Clinical data
[x]	[ ] Dual use research of concern
[ ]	[x] Plants

Methods

n/a	Involved in the study
[x]	[ ] ChIP-seq
[ ]	[x] Flow cytometry
[x]	[ ] MRI-based neuroimaging

Plants

Seed stocks

Guillardia theta CCMP2712 culture (wild type) was obtained from the Bigelow National Center for
567 Marine Algae and Microbiota.

Novel plant genotypes

Describe the methods by which all novel plant genotypes were produced. This includes those generated by transgenic approaches, gene editing, chemical/radiation-based mutagenesis and hybridization. For transgenic lines, describe the transformation method, the number of independent lines analyzed and the generation upon which experiments were performed. For gene-edited lines, describe the editor used, the endogenous sequence targeted for editing, the targeting guide RNA sequence (if applicable) and how the editor was applied.

Authentication

Describe any authentication procedures for each seed stock used or novel genotype generated. Describe any experiments used to assess the effect of a mutation and, where applicable, how potential secondary effects (e.g. second site T-DNA insertions, mosiacism, off-target gene editing) were examined.

Flow Cytometry

Plots

Confirm that:

- [x] The axis labels state the marker and fluorochrome used (e.g. CD4-FITC).
- [ ] The axis scales are clearly visible. Include numbers along axes only for bottom left plot of group (a 'group' is an analysis of identical markers).
- [x] All plots are contour plots with outliers or pseudocolor plots.
- [x] A numerical value for number of cells or percentage (with statistics) is provided.

Methodology

Sample preparation

For the Gt-ABs enrichment, whole cultures were subjected to the following filtration/centrifugation procedure: 10–15 mL of culture was vacuum filtered at very low pressure in 47 mm all glass filtration system (Millipore), using Munktell Filtrak™ Grade 1F Qualitative High Purity Lab Filter Papers (Ahlstrom-Munksjo, pore size 5–6 µm). In the filtering flask, the recovered liquid (Gt-ABs and cells of small diameter), was mixed with 30–35 mL of Milli-Q® to wash the culture media from the samples. The filtration and washing procedure were repeated once more. All the volume was transferred to F50 tubes for centrifugation at 20 degrees, 3220 rcf for 10 minutes to concentrate the Gt-ABs. Most of the supernatant was discarded and 2 ml of supernatant was left in the F50 to resuspend the pellet. The sample was transferred to 2 ml Eppendorf tubes and centrifuged at 7800 rcf for 3 minutes, where a small white pellet with the Gt-ABs was collected 606 after centrifugation. Small aliquots of the samples were examined under the light microscope (40X standard objective) between each centrifugation step to ensure the proper collection of the Gt-ABs. The enriched Gt-ABs samples were fixed with 1% PFA and permeabilized. Following permeabilization, samples were stained with DAPI (4′,6-diamidino-2-phenylindole, 50 µg/mL), incubated for 30 minutes at RT in the dark and washed twice with PBS. Pellets were resuspended in 70% FACS Flow buffer (BD Bioscience, San Jose, CA, USA) and filtered through 40 µm Flowmi Cell strainer (SP Bel-Art, Wayne, NJ,
719 USA) just before analysis and sorting.

Instrument

BD FACS Aria III flow cytometer equipped with four lasers: violet (405 nm), blue (488 nm), yellow-green (561 nm) and red (633 nm) lasers (BD Biosciences, San Jose, CA, USA)

Software

The BD FACSDiva software version 8.0.3 was used for data analysis.

Cell population abundance

The purity of the sorted sample was asessed with Confocal microscopy.

Gating strategy

Detection and recording of the Gt-ABs was done based on the sample's optical properties (forward scatter (FSC) and side scatter (SSC)). The FSC was initially filtered through a 1.0 neutral density filter and then perceived by a photodiode detector with a 488/10 nm bandpass filter. Functional analysis was performed via chlorophyll and general auto fluorescence as well as DAPI fluorescence excited by blue and yellow-green laser and violet laser, respectively. Autofluorescence emission spectra were perceived by 695/40 filter with 655 long pass (LP) mirror and 780/60 filter with LP mirror while DAPI fluorescence was detected in intervals 450/40 nm and 610/20 nm with 595 nm LP mirror. The relevant size estimation of the suspension particles analyzed was facilitated by

calibration beads with known diameters (2, 3 and 6 µm; BD biosciences). Purification of Gt-ABs from G. theta cells (size 5– 14 µm) remaining in the enriched samples was possible due to their relative size difference and chlorophyll content.

A sequential gating strategy led to the selection of distinct Gt-ABs subpopulations (Figure 3). Firstly, the identified population of G. theta cells with chlorophyll autofluorescence emission and relative size larger than 5 µm was excluded from sorting. Gt-ABs were then identified in the subsequent population based on their relative size (2–5 µm). Within this subpopulation of Gt-ABs, DAPI-positive and -negative were separately selected after comparison with the respective population of DAPI negative Gt-ABs in the unstained control samples. Four subpopulations were identified and sorted: small DAPI-positive Gt-ABs (relative size c.a 2 µm), small DAPI-negative Gt-ABs, large DAPI-positive Gt-ABs (relative size c.a 3–5 µm) and large DAPI-negative Gt-ABs.

☒ Tick this box to confirm that a figure exemplifying the gating strategy is provided in the Supplementary Information.
